# Improved understanding of the respiratory drive pathophysiology could lead to earlier spontaneous breathing in severe acute respiratory distress syndrome

**DOI:** 10.1097/EA9.0000000000000030

**Published:** 2023-08-24

**Authors:** Fabrice Petitjeans, Sandrine Leroy, Cyrille Pichot, Marco Ghignone, Luc Quintin, Dan Longrois, Jean-Michel Constantin

**Affiliations:** From the Critical Care, Hôpital d’Instruction des Armées Desgenettes, Lyon, France (FP, LQ), Environmental Justice Program, Georgetown University, Washington, DC (SL), Hôpital Louis Pasteur, Dole (CP), Université de Paris (Diderot, Sorbonne), Hôpital Bichat and UMR 5698 and GRC 29, DMU DREAM (DL), Hôpital Pitié-Salpêtrière, Paris, France (J-MC) and JF Kennedy North Hospital, West Palm Beach, Florida, USA (MG)

## Abstract

Optimisation of the respiratory drive, as early as possible in the setting of severe acute respiratory distress syndrome (ARDS) and not its suppression, could be a new paradigm in the management of severe forms of ARDS. Severe ARDS is characterised by tachypnoea and hyperpnoea, a consequence of a high respiratory drive. Some patients require endotracheal intubation, controlled mechanical ventilation (CMV) and paralysis to prevent overt ventilatory failure and self-inflicted lung injury. Nevertheless, intubation, CMV and paralysis do *not* address *per se* the high respiratory drive, they only suppress it. Optimisation of the respiratory drive could be obtained by a multimodal approach that targets attenuation of fever, agitation, systemic and peripheral acidosis, inflammation, extravascular lung water and changes in carbon dioxide levels. The paradigm we present, based on pathophysiological considerations, is that as soon as these factors have been controlled, spontaneous breathing could resume because hypoxaemia is the least important input to the respiratory drive. Hypoxaemia could be handled by combining positive end-expiratory pressure (PEEP) to prevent early expiratory closure and low pressure support to minimise the work of breathing (WOB). ‘Cooperative’ sedation with alpha-2 agonists, supplemented with neuroleptics if required, is the pharmacological adjunct, administered immediately after intubation as the first-line sedation regimen during the multimodal approach. Given relative contraindications (hypovolaemia, auriculoventricular block, sick sinus syndrome), alpha-2 agonists can help attenuate or moderate fever, increased oxygen consumption VO_2_, agitation, high cardiac output, inflammation and acidosis. They may also help to preserve microcirculation, cognition and respiratory rhythm generation, thus promoting spontaneous breathing. Returning the physiology of respiratory, ventilatory, circulatory and autonomic systems to normal will support the paradigm of optimised respiratory drive favouring early spontaneous ventilation, at variance with deep sedation, extended paralysis, CMV and use of the prone position as therapeutic strategies in severe ARDS.

**GLOSSARY:**

Glossary and Abbreviations_SDC

**KEY POINTS**

- The conceptual frame is that ARDS mortality is related more to inflammation, acidosis or high inspiratory muscle activity than to poor oxygenation.
- In contrast to suppression of the respiratory drive through mechanical ventilation, deep sedation and paralysis, management of severe ARDS should also consider optimisation of the respiratory drive, immediately following endotracheal intubation and mechanical ventilation, thus promoting early spontaneous breathing. This could be obtained by individually addressing each input to the respiratory generator.
- Given that hypoxaemia is one of the least potent inputs to the respiratory drive, once most of the other inputs to respiratory drive have been managed, spontaneous breathing (pressure support) under cooperative sedation could minimise ventilator-induced lung injury and address severe hypoxaemia.

## Introduction

Severe acute respiratory distress syndrome (ARDS) is defined by *P*_a_O_2_/FiO_2_ (P/F) less than 100, positive end-expiratory pressure (PEEP) at least 5 cmH_2_O, diffuse bilateral opacities without cardiogenic pulmonary oedema or fluid overload.^1–3^ This manuscript extends a previous viewpoint,^4^ which discussed the potential advantages of early spontaneous breathing in ARDS management. The questions addressed in this manuscript are: can appropriately managed, early spontaneous breathing improve outcome in the setting of early severe ARDS, as opposed to the current ‘state-of-art’ therapeutic strategy of controlled mechanical ventilation (CMV: ‘passive ventilation’, i.e. paralysis+proning); which sedative should be used to promote early spontaneous breathing?

Managing early severe diffuse ARDS requires taking into consideration the intensity of the activity of the inspiratory muscles (’respiratory drive’).^5–7^ A high respiratory drive leads to high tidal volume (*V*_t_, ‘hyperpnoea’), increased respiratory rate (RR, ‘tachypnoea’)^8^ and increased transpulmonary end-inspiratory pressure (stress).^9–11^ A high respiratory drive aggravates preexisting lung injury, setting the patient into a vortex^12^ [self-inflicted lung injury (SILI)].^13^ The more severe the lung injury, the higher the drive.^7^ Thus, when noninvasive ventilation (NIV) does not reduce transpulmonary pressure swings,^14^the persistence or increase in laboured breathing^15^ results in the need for endotracheal intubation with CMV and paralysis (i.e. controlled mandatory ventilation) to break-up overt ventilatory failure and the vicious circle of high *V*_t_, work of breathing (WOB), drive and SILI. The use of NIV^16^ or delaying invasive ventilation before intubation is not further considered in this manuscript.

## Controlled mechanical ventilation vs. spontaneous breathing

### Limitations in state-of-the-art

Intubation+CMV+paralysis^17^ and prone positioning (’proning’)^18^ may not be the only way to manage ARDS. Any discussion on the potential benefit of spontaneous breathing in ARDS needs a reappraisal of the indications and side effects of mechanical ventilation. Thus, intubation is indicated on clinical signs and severity of the disease (higher sequential organ failure assessment score (SOFA), immunodepression, lower P/F) rather than measures of ventilator profiles (e.g. high *V*_t_ or respiratory rate) or SILI.^19^ Shortened paralysis is a function of the intensity of the inputs (severity of ARDS; agitation, fever and source control, and renal replacement therapy). During stabilisation of the acute cardioventilatory distress,^20^ mechanical ventilation is a life-saving intervention;^19^ However, “Mechanical ventilation (in and of itself) does not produce lung healing”.^21^ Side-effects of the deep sedation/paralysis required to implement CMV are cognitive (delayed emergence, emergence delirium) and muscular impairment.^22,23^ Furthermore, CMV *per se* induces ventilatory [volutrauma and atelectrauma; ventilator-induced lung injury (VILI)], circulatory, and innate immune dysfunctions. Optimisation of CMV has not been consistently associated with improved outcome. For instance, *V*_t_ = 6 ml kg^−1^ reduces mortality by 22%.^24^ However, there are no data showing any superiority of 2 vs. 4 vs. 6 ml kg^−1^, combined or not with venovenous CO_2_ removal. Indeed, mortality is a linear function of the driving pressure below 14 cmH_2_O.^25,26^ Similarly, proning reduces dramatically the mortality evoked by moderate and severe ARDS.^18^ Nevertheless, no upright^27^ control was used in the pivot trial.^18^ Other limitations of CMV in severe ARDS are worth mentioning.

CMV+paralysis+proning do not address the pathophysiology, that is, early expiratory airway alveolar or bronchiolar^28^ closure (’closure’). They are only rescue therapies but do not heal the lung:^21,29^ “Loss of muscle tone, as caused by muscle relaxants, anaesthetics, and sedatives, and the use of high oxygen concentration in inspired gas are the prerequisites to produce atelectasis in the healthy lung during anaesthesia. This is common treatment in ARDS and certainly adds to the collapse and consolidation caused by the disease itself. Maintenance of muscle tone and modest use of supplemental oxygen may be a better approach to treatment than abuse of muscle depressants and oxygen^30^.” Closure^31^ is a consequence of compressive atelectasis (alveolar collapse) or diffuse alveolar disease.

Paralysis lowered the mortality of ARDS patients,^17^ presumably by minimising dyssynchrony, thus inflammation.^32^ Nevertheless, the duration of paralysis is controversial. For example, a 48 h duration of paralysis was selected initially only to homogenise the clinical practice of intensive care units (ICU) involved in a large trial.^17^ Later, the same group proposed to reduce this interval to 24 h,^33^ and 12 h was also considered. However, irrespective of COVID ARDS or typical ARDS, in our clinical practice we often observe prolonged paralysis for days on end.

The duration of the paralysis should, therefore, be individualised. Although the life-saving effect of CMV is not debated, weaning should be addressed within 24 h of CMV.^21^ The reason for early consideration of weanability relates to heterogeneity of ARDS. Some ARDS patients recover quickly.^34,35^ Usually, isolated pulmonary disease (e.g. aspiration) allows for normalisation of the respiratory drive within 3 to 6 h. By contrast, under extracorporeal membrane oxygenation (ECMO), 30% of the patients do not recover quickly and cannot withstand spontaneous breathing trials,^36^ with inflammation as the limiting factor.^36^ The high respiratory drive observed in COVID ARDS requires stringent normothermia (35 < *Θ* < 36 °C) and high doses of alpha-2 agonist.^37^ When major metabolic acidosis occurs (e.g. abdominal sepsis^38^), a longer interval before considering a spontaneous breathing trial is required. Only repeated assessments of a normalisation of the respiratory drive, and associated weanability,^21^ allow one to infer some lung healing and, possibly, a switch to spontaneous breathing. Importantly, no control group with an appropriate spontaneous breathing protocol^39–42^ was included in the pivot trial.^17^

**Fig. 1:**
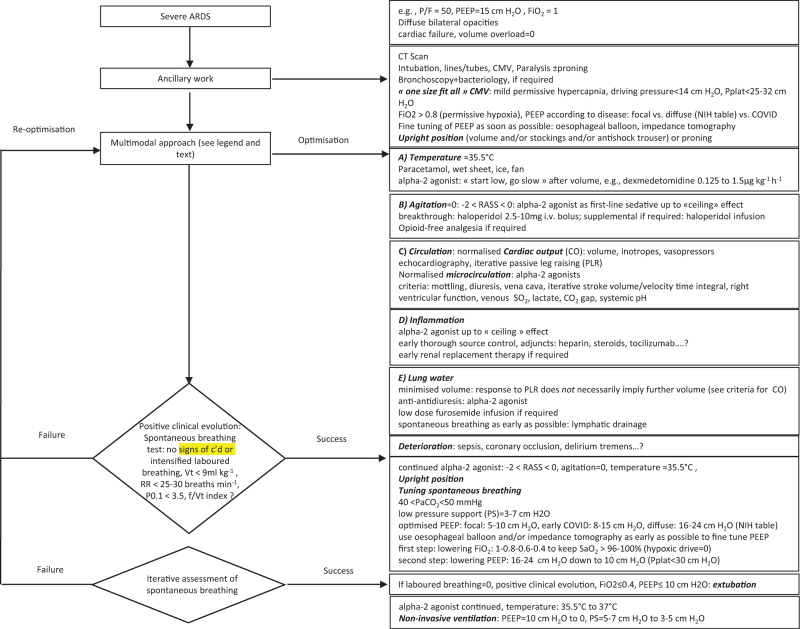
Sequential use of conventional management then multimodal approach to normalise the respiratory drive and to achieve early spontaneous breathing in early severe diffuse ARDS.

By analysing the patient's status before and immediately after endotracheal intubation/paralysis, the various inputs/stimuli leading to high inspiratory activity should be managed one after the other. Additional interventions (insertion of tubes, catheters, imaging, fibreoptic bronchoscopy; setting up the driving pressure,^25^ PEEP^43–45^ and high FiO_2_: Fig. 1) allows one to start early ‘normalisation’ of the respiratory drive (e.g. control of fever and agitation). The present reinterpretation of previous data leads to a ‘multimodal approach’^46^ (’analytical management’^47,48^). The sequence is first CMV+paralysis+proning, then a *race* against the side-effects of passive ventilation immediately following intubation to normalise the inputs to the respiratory drive (Fig. 1) and third early spontaneous breathing, as soon as^49–51^ some improvement is observed.

Given a dire clinical situation (P/F∼50, PEEP = 15, FiO_2_ = 1), intubation, controlled mandatory ventilation (CMV) and paralysis are expedited. Proning is used early given compressive atelectasis. CMV is set using a “one size fit all” approach (driving pressure <14 cm H_2_0; PEEP according to the NIH table and the disease: focal *vs*. COVID *vs*. diffuse: 5 *vs* 8–10 vs 16 cm H_2_0 respectively;^11,58,155,161^ Pplat <25–32 cm H_2_O; FiO_2_ = 0.8 to 1.^19^ Immediately following intubation, optimisation begins, using Eq. (1) as a checklist: (*Vt,RR*)=*f(temperature, agitation, inflammation, lung water, cardiac output [CO]/blood pressure [BP]/microcirculation/systemic pH, PaCO*_*2*_*, PaO*_*2*_):

1) Temperature^126^ is lowered to 35<θ<36°C. An alpha-2 agonist is infused up to ceiling effect^197^ (e.g., dexmedetomidine 1.5 μg kg^−1^ h^−1^; start low, go slow). To accelerate cooling, one administration of paracetamol is combined with a wet sheet+fan or Bair Hugger®.

2) Agitation is controlled to stringent quietness (−2<RASS<0) with the alpha-2 agonist.^108,110^ Breakthrough is addressed with haloperidol bolus (2.5–10 mg i.v.). If required haloperidol infusion is set (50 mg in 48 ml saline: 0.2–2 ml h^−1^).

3) Cardiac output (CO) is optimised. Volume load is administered before intubation to preempt increased intrathoracic pressure caused by CMV,^198^ Iterative passive leg raising (PLR) allows one to administer volume up to normalisation of the clinical pattern:^97^ capillary refill time, mottling, diuresis, venous SO_2_, CO_2_ gap, systemic pH, velocity-time integral, vena cava. A positive BP or CO response to PLR does not automatically imply further volume administration, based primarily on the clinical pattern. The right ventricle (and a possible foramen ovale^82^) is iteratively assessed. Vasopressors and inotropes are administered if required. Microcirculation^92,93^ is restored over 3–12 h thanks to the normalisation of the vascular sympathetic activity evoked by the alpha-2 agonist.

4) Thorough early source control is mandatory. Inflammation is addressed using the indirect anti-inflammatory effect of the alpha-2 agonist.^115–123^ Adjuncts are used if required, including early renal replacement therapy to lower plasma concentration of inflammatory mediators.

5) Lung water is minimised by reducing the volume load, using the anti-ADH effect of the alpha-2 agonist^99–103^ and if necessary low dose furosemide infusion.

6) “one size fit all” ventilation is individualised as early as possible under CMV: oesophageal balloon^34,139^ to suppress atelectrauma, inflammation (self- or ventilator induced) and bronchial closure; impedance tomography.

7) Upright position (reverse Trendelenburg, 60° head up, 45° leg down).^6^

As soon as^48^ some clinical improvement is observed, paralysis is withdrawn. Spontaneous breathing (SB) begins using low pressure support∼7 cm H_2_0, low inspiratory trigger, 100% automatic tube compensation^172^ and low expiratory trigger.^166^ To take advantage of SB, PEEP is increased if required by the disease (above).

SB should be achieved without laboured breathing or any sign of impending ventilatory or circulatory failure (sternomastoid contraction, tracheal tug, suprasternal notch retraction, intercostal recession, diaphoresis,^199^ thoraco-abdominal swings; capillary refill time, mottling, diuresis, enlarged CO_2_ gap, lowered venous saturation, lactate). Should laboured breathing reappear any time after SB, a) causes of deterioration are assessed (sepsis, myocardial, delirium); b) the multimodal approach begins again; c) paralysis is readministered immediately.

Given an improved clinical pattern (including chest X ray, markers of inflammation, etc.), FiO_2_ is reduced step by step to 0.4 with SaO_2_>96 (hypoxic drive=0). Once FiO_2_ is 0.4, then PEEP is lowered step by step to ≤10 cm H_2_0. If the clinical pattern is improved (no laboured breathing, adequate CO, diuresis and markers of inflammation, P/F>150, FiO_2_≤0.4, PEEP≤10 cm H_2_0), the trachea is extubated. Continuous noninvasive ventilation is set under continued alpha-2 agonist administration to RASS<0. PEEP is lowered to zero PEEP. Temperature increases slowly to 37°C.

#### Deep vs. light sedation

Deep sedation is required^52^ in the setting of severe ARDS, defined here as FiO_2_ = 1, PEEP = 15 and P/F ∼50 and acidosis. This acidosis occurs in the sickest patients, ∼7 to 11% of the patients. However, alternative strategies have been proposed.^53^ Deep sedation leads too often to *de facto* general anaesthesia.^54^ Deep sedation is associated with reverse triggering,^32^ more severe circulatory side-effects^55^ and perhaps mortality in some studies.^56^ The trend toward lighter sedation observed before COVID^32,55^ was reversed to address a very high drive observed in COVID-ARDS. In this respect, most hold that there is no simplistic treatment other than sedatives and opiates for controlling the myriad of inputs to the respiratory generator (’input’). Conservatively, paralysis is used in the presence of high drive or breath-stacking.^32^ In this respect, the high drive observed in the setting of COVID was suppressed by passive hyperventilation below the apnoea threshold^57^ (sedation withdrawal, lowered PEEP and early recovery^57^) and questions the use of deep sedation. As sedation is an unsettled question, we suggest using ‘cooperative sedation’ based on alpha-2 agonist (dexmedetomidine or clonidine) to avoid, where possible, deep sedation.

### Spontaneous breathing: arguments against

The use of spontaneous breathing in severe diffuse ARDS remains unaddressed in an analysis,^58^ as spontaneous breathing patients were less sick than CMV patients.^58^

(1) Ventilatory muscles: decompensated chronic obstructive pulmonary disease (COPD) patients need resting their muscles up to several days.^59^ In contrast, most ARDS patients present with intact ventilatory muscles upon admission to the ICU. Would muscle resting and paralysis be unnecessary? We need caution when examining this hypothesis: on day1 in the ICU, 64% of the patients, with or without ARDS, presented a reduced capacity of the diaphragm to generate inspiratory pressure;^60^ complete recovery of septic or fatigued ventilatory muscles may require up to 48 h.^59^ Thus, when continued laboured breathing^15^ leads to failure, intubation and CMV are needed.^59^
(2) Lung: the argument against spontaneous breathing is the worsening of a high *V*_t_ and the SILI vicious circle:^12,62^ opening/closing (atelectrauma), pendelluft and inflammation reinforce the vicious circle.^12^ Indeed, the ARDS lung is a restrictive lung, which cannot withstand the large *V*_t_ generated by intact muscles. By contrast, lowering the drive minimises *V*_t_ and SILI, and vice versa.

In animals, sodium salicylate injected in the cisterna magna evokes hyperventilation and death: these are avoided by barbiturates and paralysis.^61^ However, deep sedation and paralysis are not the treatment of hyperventilation, as stated (’one could imagine other means in the future’^13^). The activity of the respiratory generator is not normalised back to baseline by barbiturates and paralysis. Pharmacologically, centrally, the generator is suppressed not normalised; peripherally, the ventilatory consequences on muscles and lung are suppressed. As the respiratory generator and ventilatory muscles are fully blunted by deep sedation and paralysis, they do not offer indication of the inputs anymore and their improvement or worsening is masked. This leaves the pathophysiological causes of the increased drive unchanged, for example, following emergence from deep sedation.^63^

### Spontaneous breathing: pros

Side-effects of CMV suggest using only short, upfront paralysis and early assessment of weaning.^21^ To offset a vicious cycle of prolonged CMV+paralysis, the respiratory drive should be referred to its inputs. Indeed, *V*_t_ and respiratory rate (RR) are functions of pH, *P*_a_CO_2_, *P*_a_O_2_,^64^ temperature,^65^ ‘neural drive’,^6,7,66^ inflammation,^36^ increased permeability^67^ and lung water,^68^ as in Equation 1: *(V*_*t*_*,RR)  = f[temperature, agitation, inflammation, lung water, cardiac output (CO)/blood pressure (BP)/microcirculation/systemic pH, P*_*a*_*CO*_*2*_*, P*_*a*_*O*_*2*_*]*, a check list useful when managing a patient, at 03 am on call. Based on our current clinical practice and the published data from 51 patients,^37,69^ we suggest early spontaneous breathing as an alternative to mandatory ventilation and paralysis.

## Pathophysiology

The matter is to return the functioning of the circulatory, respiratory, ventilatory and autonomic systems to baseline, in a hierarchised manner (i.e. so-called integrative physiology). The pathophysiology of the respiratory drive suggests a hierarchical description of the different inputs.

### Oxygen, a weak stimulus

O_2_ is ∼20 times less diffusible than CO_2_. This explains the requirement for increased expiratory surface area for O_2_ diffusion. The diffuse alveolar damage should heal by itself, unaggravated by mechanical ventilation (’buying time’ ^70^). Didactically, hypoxaemia is separated from the other inputs (Equation 1).

The objective is not hyperoxaemia but suppressing the hypoxic drive (SaO_2_ ≥96%) and increased RR and spontaneous *V*_t_. Given the shape of the oxygen haemoglobin dissociation curve (saturation vs. partial pressure), an SaO_2_ at least 96% corresponds to a *P*_a_O_2_ anywhere between 60 and 200 mmHg.^21^ Caution is needed as the carotid bodies respond to *P*_a_O_2_, but not to SaO_2_,^29^ requiring iterative blood gases.

(1) Peripheral chemoreceptors are relatively insensitive to mild hypoxaemia^66^ and generate an ‘independent weak’^66^ unsustained^71^ response. Indeed, the minute ventilation for a maximal stimulus is much higher for H^+^ and CO_2_ as opposed to O_2_^64^ (weak peripheral chemoreflex to O_2_ vs. strong central chemoresponse to H^+^ and CO_2_). Acute hypoxaemia evokes brief hyperventilation only at low *P*_a_O_2_, followed by an ‘hypoxic ventilatory decline’:^72^*P*_a_O_2_ less than 55 mmHg^64,66^) and hyperventilation is reduced with age.^64^
(2) Hyperventilation is observed when *P*_a_O_2_ is less than 55 mmHg and reduced in the setting of hypocapnia.^64^ An end-tidal *P*_a_CO_2_ less than 29 mmHg (*P*_a_CO_2_ ∼34 mmHg) prevents the hypoxic ventilatory response even if SaO_2_ is 70% or less:^72^ ∼30% of healthy individuals do not experience dyspnoea despite severe hypoxaemia (*P*_a_O_2_ = 40 mmHg, *P*_a_CO_2_ = 40 mmHg).^72^ Patients present low SaO_2_ without tachypnoea or hyperpnoea in the setting of SARS-CoV2-evoked ARDS (COVID-ARDS; ‘silent hypoxaemia’)^73^: there is no causal relationship between hypoxaemia and dyspnoea.^72^ Low SaO_2_ requires close monitoring but not necessarily intubation and mechanical ventilation, to be expedited on the clinical pattern (continued or intensified vigorous laboured breathing,^15^ pulmonary infiltrates with abnormal gas exchange or increased WOB^21^), but low SaO_2_.
(3) Hypoxaemia has minimal effect by itself, but it does alter responses to acidosis.^6,7,66^ Therefore, addressing systemic acidosis and hypercapnia are priorities. Hypoxaemia is the next priority with respect to ventilatory stimuli. Indeed, in the setting of noninvasive ventilation, reduction of transpulmonary pressure swing is not a function of improved oxygenation and increased P/F.^14^ In the setting of invasive ventilation, outcome is not associated with improved oxygenation^74–76^ but is with reducing driving pressure.^25^
(4) In the healthy normocapnic volunteer, increasing *P*_a_O_2_ from 40 to 100 mmHg lowers the minute ventilation from 20 to 5 L.min^−1^.^66^ Accordingly, in the setting of late ARDS, low SaO_2_ is followed by increased RR; conversely, increased SaO_2_ is followed by decreased RR.^77^

### Acidosis, CO_2_, neural drive, inflammation

All the inputs should be immediately and simultaneously addressed during the few golden hours following intubation, using a hierarchised approach: respiratory generator, ventilatory muscles, lung tissue and lung receptors. A window of opportunity exists to break down the drive to the components of Equation 1. Marathoner runners address severe acidosis after the finish line by cooling, resting and hydrating. Climbers face severe hypoxaemia, low lactate and alkalosis^78^ and diving mammals excrete CO_2_ and metabolise high lactate quickly allowing them to repeat dive within a short time, due in part to relaxed venoconstriction.^79^ Nevertheless, in contrast with trained athletes, ARDS patients cannot manage acidosis, hypercapnia and lactate by themselves. Is this caused by inadequate microcirculation or inadequate handling of lactate or prolonged inflammation?

#### Acidosis

##### Circulation

In the setting of circulatory distress, the WOB is much higher under spontaneous breathing vs. CMV. Respectively, 21 vs. 3% of CO is diverted to the diaphragm.^80^ Thus, CMV+paralysis breaks up a vicious cycle, redirects blood flow away from the diaphragm and improves systemic circulation. However, paralysis does not heal the lung.^21,29^ Immediately before^81^ and following CMV+paralysis, CO^82^ should be optimised. First, this optimisation will increase a low PvO_2_ effect,^83,84^ normalise intrapulmonary shunting and avoid a pseudonormalised shunt.^85^ Second, the possibility of a foramen ovale being present with additional cardiac right-to-left shunting needs to be addressed as an additional cause of hypoxia, which may be present in ∼20% of the patients.^86^ Third, improved CO will help normalising systemic acidosis. Fourth, the use of vasopressor will increase the right coronary perfusion pressure,^87^ and help addressing RV failure.^88^ In the setting of shock, vasopressors will normalise BP. In turn, normalised baroreceptors will no longer stimulate the respiratory drive and the cardiac and vascular sympathetic system. This will improve the micro-circulation. Fifth, spontaneous breathing allows for diaphragmatic movements. They squeeze the hepato-splanchnic blood^89^ and increase the venous return. To sum up, adequate CO is the *prerequisite* to address ARDS. The cornerstones of ARDS management^90^ are CO combined with ‘best PEEP’ set on the highest compliance.

##### Metabolic acidosis

In patients, acidosis prevents switching to spontaneous breathing.^38^ Thus, acidosis is key in controlling hyperpnoea. Spontaneous breathing requires stringent criteria. First, 9% of patients cannot switch to spontaneous breathing under sevoflurane when the pH is less than 7.20 (septic or cardiogenic shock, opiate overdose).^91^ Second, spontaneous breathing with a pH = 7.28 has been associated with pendelluft.^92^ Nevertheless, one case report cannot lead to the rejection of spontaneous breathing for all patients in the setting of early diffuse ARDS, after correction of acidosis. A pendelluft is minimised with high PEEP, which makes spontaneous breathing less injurious.^93^

Indeed, pH seems paramount when switching to spontaneous breathing: 9% of patients cannot switch to spontaneous breathing under sevoflurane.^91^ In contrast, successful spontaneous breathing under sevoflurane is associated with a pH = 7.32 ± 0.11.^94^ Presumably, what applies to COPD patients applies also to switching to spontaneous breathing following normalisation of pH (e.g. >7.23 to 7.29 or >7.32 ^36,94,95^, to be addressed).

Restored microcirculation^96–98^ normalises skin and vasomotor sympathetic hyperactivity, inflammation (‘metaboreflex’^99^) and the elevated drive, which persists following correction of systemic pH. Optimised circulation allows one to switch to spontaneous breathing as soon as the clinical pattern improves.^49^

##### Respiratory acidosis

Hypocapnia leads to apnoea in healthy volunteers.^6^ This contrasts to early ARDS (*P*aCO_2_ ∼32 mmHg with high *V*_t_: *V*_t_ ∼11 ml kg^−1^).^8^ Hypocapnia observed before intubation^8^ converts to hypercapnia following intubation, paralysis with low *V*_t_. High-normal carbon dioxide partial pressure (40 < *P*_a_CO_2_ < 50 mmHg) should be achieved before switching to spontaneous breathing, using fever and agitation control (lowered VO_2_). An alpha-2 agonist suppresses the hyperventilation observed during mild hypercapnia in the setting of spontaneous breathing in healthy volunteers^100^ and also under spontaneous breathing and cooperative sedation in ARDS patients.

##### Lung water

As alveolar oedema leads to massive intrapulmonary shunt,^85^ minimising filling pressure^82^ and lung water is necessary. First, iterative passive leg raising (PLR) minimises volume load.^101^ CO or BP response to PLR does *not* necessarily imply further loading, based only on the clinical improvement delineated above. Second, spontaneous breathing allows lymphatic drainage to operate fully, in contrast to CMV+paralysis.^102^ Third, alpha-2 agonists evoke Na^+^ and water diuresis (anti-ADH).^103–107^ Fourth, sympathetic blockade reduces pulmonary venous pressure and lung oedema.^108^

#### Neural drive

##### Neural drive

The baseline neural drive^6,7^ is partially independent of H^+^ and CO_2_.^10^ It increases during alertness, pain, exercise,^64^ anxiety and agitation.^109,110^ Neural drive may be very high in the setting of ARDS under ECMO^10^ linked to inflammation.^36^ Despite removal of ∼80% VCO_2_ and normalised pH, *P*_a_O_2_, and *P*_a_CO_2_, high transpulmonary inspiratory pressure (∼38 cmH_2_O) was observed.^10^ Similarly, under ECMO, a high gas flow can reduce CO_2_ but does not produce apnoea nor lower RR^36^*per se*.

##### Sedation

Benzodiazepines and opiates depress the respiratory generator, hindering spontaneous breathing. By contrast, as alpha-2 agonists preserve the activity of the respiratory generator^111^ and we believe they should be first-line sedatives administered immediately following intubation.^112–115^ Contraindications are sick-sinus syndrome, atrioventricular block, or hypovolaemia (which needs to be corrected before administration alpha-2 agonist).

#### Fever control and inflammation

Injection of endotoxin increases the drive, independent of fever or symptoms.^116^ Thus, source control is paramount. In the context of late ARDS, low *V*_t_ is observed (rapid shallow breathing^117^). Spontaneous breathing cannot then be sustained because of factors independent from pH or *P*_a_CO_2_.^10^ Inflammation may be the main contributor to the drive:^36^ does prolonged lung inflammation preclude spontaneous breathing? Conversely, does ECMO increase systemic inflammation evoking an inability to spontaneous breathing?

Pulmonary, central nervous system (CNS) or systemic inflammation are to be separated. CNS inflammation increases the central hypoxic response despite adequate control of systemic hypoxaemia:^118^ a selective enhancement of hypoxic drive was observed in absence of arterial hypoxaemia.^118^ This makes the control of CNS inflammation more difficult. Systemically, alpha-2 agonists normalise sympathetic hyperactivity leading to an indirect anti-inflammatory effect: lowered proinflammatory cytokines and procalcitonin, increased anti-inflammatory cytokines.^119–127^ Alpha-2 agonists improve obstructive disease,^128^ of relevance as intrinsic PEEP is common in ARDS.^129^

Improved outcome following fever control is observed, including in the setting of ARDS (∼50% of the patients included in trial^130^). As a restrictive ‘baby’ lung can accommodate only a minimised VO_2_, ‘reducing metabolic demand [is] among the most important unproven rules [of] management’.^131^ When the neural drive is high, as in COVID ARDS, early fever control to ∼35.5 °<θ<36°C^132^ coupled to high-dose alpha-2 agonist^100,133–135^ is key to avoid deep sedation, paralysis and prolonged intubation.^37^

## Ventilatory settings

Given the pathophysiology of ventilation during spontaneous breathing, it is important to delineate the parameters of the ventilatory support.

### High positive end-expiratory pressure and lung geometry

Under spontaneous breathing, altered gas exchange is a consequence of alveolar hypoventilation (not considered here) or VA/*Q* mismatch (COPD) or intrapulmonary shunt ('shunt’).^136^ Shunt is the primary mechanism of hypoxaemia in ARDS; nevertheless, low ventilation/perfusion ratio (VA/*Q*) areas also exist (partially filled alveoli, increased airway resistance).^136^ If cardiac output is normalised,^85^ PEEP decreases shunt and redistributes flow from nonventilated and shunt (VA/*Q* = 0) areas to increased or normal VA/*Q* areas (reopening of collapsed alveoli with decreased shunt).^85,136^

The issue is not to fully revert alveolar collapse^137,138^ (’open up the lung’^139^). By contrast, in the present management, PEEP targets only low VA/*Q* areas (’poorly aerated lung’)^140^ or bronchial closure.^28^ Under spontaneous breathing, active diaphragmatic brake prevents early alveolar closure^141^ and improves VA/*Q*.^41,142^ Given the need to limit plateau pressure^43–45,143^ under CMV, achieving high PEEP requires lowering *V*_t_. The drawbacks are possible hypercapnia and right ventricular (RV) failure.^144^

In the literature, the commonly accepted values are: plateau pressure = 25 to 32 cmH_2_O^43–45,143^; driving pressure less than 14 cmH_2_O^25^ measured during inspiratory hold.^145^ A successful switch from CMV+paralysis to spontaneous breathing allows one to observe a lowered plateau pressure (30 to 25 cmH_2_O^146^) and a lowered transpulmonary driving pressure (13 to 12 cmH_2_O).^147^ In the setting of low compliance and CMV, a high driving pressure is observed. Therefore, given a limited plateau pressure, a high PEEP can be achieved only if driving pressure is lowered. This implies switching to spontaneous breathing, with a maximum plateau pressure of 25 to 32 cmH_2_O.

High PEEP prevents early closure, increases expiratory time and decreases RR (Hering–Breuer reflex^148^), prevents translocation of gases from nondependent to dependent lung (’pendelluft’^92,93^), converts atelectatic lung to recruited lung ('solid’ to ‘fluid’ lung behaviour ^149^), decreases spontaneous ventilatory effort through mechanoreceptors,^7^ ensures spontaneous noninjurious inspiratory effort, lowers *V*_t_ (offsetting the need for paralysis^93^), restores the baby lung to its highest compliance^150^ and minimises the WOB^151^ (’the baby lung is small but not stiff’^152^).

### Lowering FiO_2_ with constant positive end-expiratory pressure

Under spontaneous breathing, in early severe diffuse ARDS, the hypoxic drive is to be manipulated by uncoupling^47,48,69,153^ the lowering of the high FiO_2_ vs. high PEEP. This is at variance with simultaneous changes in PEEP and FiO_2_ under CMV.^154^

Under paralysis, setting up SaO_2_ greater than 87 to 95%,^154,155^ takes advantage of the absence of tachypnoea and hyperpnea (i.e. permissive hypoxaemia). By contrast, under spontaneous breathing, a SaO_2_ at least 96% suppresses the hypoxic drive and lowers RR.^77^ In our hands, switching to spontaneous breathing allows for higher PEEP (above), then evokes a progressive increase up to SaO_2_ at least 96% over 3 to 12 h. Such high PEEP is usually needed for a short interval (12 to 72 h), compatible with slowly improving lung mechanics.^156,157^ This contrasts with a near-instantaneous lung reopening,^137,138^ during a recruitment manoeuvre. In daily practice, when FiO_2_ = 1 is briefly used, absorption atelectasis occurs^158^ and low VA/*Q* areas convert to shunt. Then, given a constant high PEEP and a steady SaO_2_ greater than 96% and spontaneous breathing, FiO_2_ is adjusted downward from 1 to 0.8 to 0.6 and finally 0.4. Under spontaneous breathing, the hypoxic drive should be always minimised.

### Lowering positive end-expiratory pressure under constant low FiO_2_

Under CMV, a combination of plateau pressure 25 or less to 32 cmH_2_O,^43–45^ driving pressure less than 14 cmH_2_O^25^ and PEEP at least 15 cmH_2_O^159^ does not necessarily recruit enough surface for O_2_ diffusion, to raise the SaO_2_ to greater than 96%, and thus suppress the hypoxic drive. By contrast, under spontaneous breathing, low pressure support and driving pressure and *V*_t_ allow one to achieve plateau pressure 30 cmH_2_O or less^43–45^ with PEEP ∼16 to 24 cmH_2_O (NIH Table^154^ or preferably measured by an oesophageal balloon), SaO_2_ greater than 96%, and a lowered FiO_2_ = 0.4 (above).

Then, under spontaneous breathing, given FiO_2_ = 0.4, PEEP is lowered step by step from ∼16 to 24^154^ to 10 cmH_2_O (decrements: 2 to 5 cmH_2_O), keeping SaO_2_ as high as possible to suppress the hypoxic drive. Arterial and venous gases address P/F and systemic and microcirculation. Any decrease in SaO_2_ to less than 96% or tachypnoea leads to increased PEEP again, to achieve SaO_2_ at least 96%. In addition, deterioration is to be addressed: lowered cardiac output, sepsis or septic shock, decompensation of delirium tremens, search for coronary occlusion, and so forth. Usually, under CMV+high PEEP, P/F improves up to at least 150 within 72 h.^34,35^ Under spontaneous breathing+low pressure support+high PEEP, P/F improves with a similar kinetics.^69,160^

The clinical pattern, the exacting control of the inputs (Equation 1), then appropriate numbers (P/F >150, FiO_2_ = 0.4 and PEEP ≤10 cmH_2_O) are compatible with extubating the trachea, followed by continuous NIV under continued cooperative sedation,^69,161,162^ then weaning.^163^

#### Schematically positive end-expiratory pressure is set twice

(1) Immediately following intubation+paralysis, the ventilator is set in a standard manner (*P*_plat_ ≤30 cmH_2_O,^45^ driving pressure <14 cmH_2_O,^25^ PEEP set with the NIH Table^154^). As soon as possible, PEEP is individualised: arithmetically, PEEP = (plateau pressure–driving pressure) is then greater than 15 cmH_2_O (diffuse ARDS ^159^). Alternatively, first, an end-inspiratory transpulmonary pressure set to 25 cmH_2_O or less^143^ allowing an increased PEEP (18 to 22 cmH_2_O; *P*_platRS_: 31 to 38 cmH_2_O). Within ∼30 min, P/F increases (67 ± 5 to 180 ± 9). The ECMO requirement is halved.^143^ Second, end-inspiratory and end-expiratory transpulmonary pressures are increased up to 25 and 10 cmH_2_O, respectively.^35^ Third, the pressure–volume or the pressure–time curves help in preventing airway collapse and and the need for PEEP.^31^ Fourth, electrical impedance is of interest to homogenise ventilation in the setting of extrapulmonary ARDS.^164^
(2) Immediately following switching to spontaneous breathing, the intrathoracic pressure is lowered.^146,147^ Given a fixed *P*_plat_, this lowered pressure allows one setting up a higher PEEP, using the NIH table or, preferably an oesophageal balloon. Four steps are used. First, CO normalisation combined to intubation+CMV+paralysis^17^ allow one to address the acute cardioventilatory distress.^20^ The lung function and oxygenation improve slowly,^156,157^ especially given the relatively low PEEP used. Second, under spontaneous breathing, a high PEEP allows one to increase oxygenation to P/F greater than 150. Third, the FiO_2_ is lowered from an FiO_2_ in the range 0.8 to 1 to 0.4 under CMV, then spontaneous breathing. Fourth, extubation is considered when the improved clinical pattern matches the numbers: FiO_2_ = 0.4, PEEP 10 cmH_2_O or less, P/F greater than 150. These values would then be suitable for a trial of NIV.

#### Low positive end-expiratory pressure

High PEEP is not considered in the setting of ‘focal’ ARDS^165^ nor in early COVID ARDS which is a different disease: pulmonary endothelial vascular inflammation, micro-thrombi or macro-thrombi, near-normal alveoli (high VA/*Q*) with high compliance (∼50 ml cm^−1^ H_2_O), poor perfusion [ventilated nonperfused (dead space) >nonventilated perfused (high shunt), high VA/*Q* mismatch^166^], loss of hypoxic vasoconstriction (hyperperfusion of gasless alveoli^167,168^), low P/F when matched for compliance (loss of hypoxic vasoconstriction and high shunt^62^). High compliance is observed with variability in recruitment (16 to 140%)^166^ or conversely little areas for recruitment.^168^ High PEEP would increase physiologic (high VA/Q areas) and anatomic dead space^136^ and *P*_a_CO_2_,^166^ as delineated in typical ARDS (pulmonary vascular injury and high dead space^169^). Therefore, in the setting of early COVID ARDS, intermediate PEEP (e.g. <10 to 15 cmH_2_O^12,168^) recruits some alveoli without redirecting blood flow away from already overstretched alveoli, that is, without increased dead space.^12^

By contrast, typical ARDS presents with diffuse alveolar damage (permeability oedema, protein-filled alveoli: hyaline membrane, lung water, inhibited surfactant as opposed to compressive atelectasis and collapsed alveoli^62^), low compliance (∼18 ml cm^−1^ H_2_O,^170^ ‘baby lung’) and nonventilated perfused alveoli (VA/*Q* = 0, ‘true’ shunt). Poor oxygenation at low PEEP may predict potential for recruitment.^166^ Indeed, compressive atelectasis is reversed by high PEEP (obesity, intra-abdominal hypertension). By contrast, high PEEP does not necessarily apply to direct diffuse alveolar damage with alveolar filling. Lastly, a high dead space in early ARDS is associated with mortality:^169,171^ under spontaneous breathing, this is relevant when a high *V*_t_ and increased WOB are present. A thoroughly normalised drive is the requisite of low inspiratory assistance, that is, the presently proposed quasi-unassisted breathing during the acute, exudative, phase of severe ARDS.

### Inspiratory assistance

Airway pressure release ventilation^41,42,53,172^ is not considered because of absence of expertise. Pressure support was delineated earlier.^47,48,173^ Low pressure support compensates the WOB caused by the valves and the circuit (3 to 5 cmH_2_O),^174^ thus lowering the circulatory burden. The complete recovery of ventilatory muscles is slow:^59^ thus, inspiratory assistance should alleviate added load to the diseased muscles. A high drive leads to high *V*_t_ independently of pressure support level.^8,175^ Hundred percent automatic tube compensation^176^ accounts for the resistance caused by the endotracheal tube (’tube’). The inspiratory trigger is set to a minimum. The pressure ramp slope is steep.^177^ The low expiratory trigger achieves the longest possible inspiratory time and reduces the ventilator-to-the-patient asynchrony^170^ (Brochard, mechanicalventilation.wordpress.com/2013/09/27). Under spontaneous breathing, achieving an appropriately high PEEP resets the lung on its best compliance (above); thus, a low pressure support is needed.^8,173,175,178,179^ Accordingly, lower pressure support–higher PEEP allows for lower FiO_2_ and higher SaO_2_.^180^ With low pressure support, the alpha-2 agonist sedation helps to avoid ventilator-to-patient asynchrony: under cooperative sedation, the pressure and flow wave forms appear monotonous without sigh or asynchrony. This contrasts with deep propofol sedation.^170,181–183^ These contrasting observations may relate to alpha-2 agonists preserving respiratory genesis.^100,111,184^

### Position

Proning^18,185^ decompresses paravertebral areas and is appropriate during short-term CMV+paralysis. Nevertheless, human adults spend 66% of their time in a vertical (or at least not horizontal) position. Does upright position lead to similar result (’upright’: reverse Trendelenburg, 60° head up, leg down 45° ^27,186–188^)? Indeed, upright increases P/F from ∼125 to ∼160 in moderate/severe ARDS, with the caveat that increased intraabdominal pressure does not occur. This occurs also under spontaneous breathing. Surprisingly, in the upright position, low BP is usually not a problem. The caveat is that quite often, ARDS patients are receiving vasopressors. Compression stockings or military antishock trouser are seldomly required: does spontaneous breathing improve venous return?^89^ Is the reactivity to noradrenaline increased by the alpha-2 agonist?^123,189–193^ Combined upright+proning^194^ and lateral decubitus may speed up weaning.

In intubated spontaneously breathing patients, *V*_t_ and RR do not necessarily reflect the inspiratory effort^58^ or the WOB. Therefore, clinical evaluation of the inspiratory muscles implies measuring an acceptable transpulmonary pressure without high *V*_t_ (iterative f/*V*_t_^195^ or oesophageal pressure swing,^14^*P* 0.1 <3.5 cmH_2_O^9,11,63,196,197–199^).

## Conclusion

In management of ventilatory failure, the underlying disorders (infection, inflammation and acidosis) lead to a high respiratory drive and should be addressed immediately following intubation. A high drive is not a boon but a bane. It is not to be suppressed but normalised to the benefit of the patient, allowing early spontaneous breathing as soon as^49^ some improvement occurs. In the setting of early, severe diffuse ARDS, early spontaneous breathing allows one to use high PEEP, the only treatment available to prevent expiratory closure, atelectrauma and inflammation, allowing the lung to heal itself. The analysis of the literature^9,30,37–42,69,91,94,95,160,200^ and the present pathophysiological approach suggest using sequentially:^48^ first CMV+paralysis+proning;^17,18,24,25^ second, a thorough normalisation of the inputs to the respiratory generator and the autonomic system (temperature, agitation, inflammation, lung water, CO/BP/microcirculation/systemic pH, *P*_a_CO_2_); third, early spontaneous breathing.

### Future challenges and unanswered questions

(1) ‘Does appropriate, early, spontaneous breathing, as opposed to CMV+paralysis+proning, improve outcome in the setting of early diffuse ARDS?’. We hypothesise that guided spontaneous breathing techniques with attention to temperature adjustment, avoiding additional iatrogenic SILI and VILI, acidosis and inflammation in reference to systemic and microcirculation are appropriate research direction to address.
(2) ‘How and when sedation and paralysis are used or titrated?’ We believe that paralysis should be used as briefly as possible while aiming to achieve normalisation of the respiratory drive. In this regard, we advocate alpha-2 agonists as first-line sedatives. Proof-of-concept data to date^37,38,69,160^ show no harm to the patient. A demonstration is required in a more usual clinical setting with high case load, and limitations of staff availability in a busy intensive care setting.

## Supplementary Material

Supplemental Digital Content
