# Supplementary material for: Improved understanding of the respiratory drive pathophysiology could lead to earlier spontaneous breathing in severe acute respiratory distress syndrome
Source: Eur J Anaesthesiol Intensive Care. 2023 Aug 24;2(5):e0030. doi: 10.1097/EA9.0000000000000030 (PMC11783659; doi:10.1097/EA9.0000000000000030)
Supplement: Supplemental Digital Content [file ejaic-2-e0030-s001.docx]

**Glossary and Abbreviations**

ARDS: acute respiratory distress syndrome.

Ataraxia: imperturbability of mind (Epicurus) (200).

BP: blood pressure

COVID-ARDS: coronarovirus2-evoked ARDS, Covid-ARDS.

CMV: controlled mechanical ventilation.

CO: cardiac output.

Cooperative sedation: sedation evoked by alpha-2 agonists (dexmedetomidine, clonidine) combining ataraxia upon absence of stimulation and alertness on stimulation.

COPD: chronic obstructive pulmonary disease.

drive: respiratory drive, intensity of the activity of inspiratory muscles, inspiratory activity.

ECMO: extracorporeal membrane oxygenation.

GA: general anesthesia.

Hering-Breuer inflation reflex: shortened duration of inspiration due to increased stretch receptor.

Inputs: stimuli impinging on the respiratory generator: temperature, agitation, inflammation, systemic pH, PaCO2, PaO2.

NIH: National Institute of Health.

PEEP: positive end-expiratory pressure.

Pendel-luft: translocation of gases from non-dependent to dependent lung.

PICO question: patient, intervention, comparison, outcome.

P0.1: airway occlusion pressure developed 100 ms after the onset of inspiration.

PPlatRS: plateau pressure of the ventilatory system.

PS: inspiratory assistance, pressure support.

P-SILI: patient self-induced lung injury.

P/F ratio: PaO2/FiO2 ratio.

proning: prone positioning.

RR: respiratory rate.

RV: right ventricle.

SB: spontaneous breathing, spontaneous ventilation.

SILI: self-inflicted lung injury

strain: lung deformation (12),Vt/end-expiratory volume ratio (58)

stress: transpulmonary pressure (58).

SsvcO2: oxygen saturation in the superior vena cava.

VA/Q: ventilation/perfusion ratio.

VE: minute volume.

Vt: tidal volume.

WOB: work of breathing.
